# Acute Cocaine Toxicity Complicated by Cerebral Edema, Respiratory Failure, and Coronary Vasospasm: A Case Report and Review of Multimodal Management Strategies

**DOI:** 10.7759/cureus.70954

**Published:** 2024-10-06

**Authors:** Jesse O'Rorke, Keri Mason

**Affiliations:** 1 Medicine, Lee Health, Fort Myers, USA; 2 Medicine, Lake Erie College of Osteopathic Medicine, Bradenton, USA; 3 Internal Medicine, Cape Coral Hospital, Cape Coral, USA

**Keywords:** acute cocaine intoxication, emergency medicine and trauma, general internal medicine, multiorgan system failure, pulmonary critical care, rhabdomyolysis

## Abstract

The use of the illicit/illegal drug cocaine is associated with many acute medical complications that often progress to chronic health conditions. These complications can affect multiple organ systems and lead to widespread organ failure at times. It is a commonly portrayed narrative in today’s society that the nefarious “lacing” of cocaine with fentanyl is the catalyst that leads to the majority of overdose deaths from this substance. While this fact is certainly true, it can often overshadow the additional important fact that a large swath of people sustain major medical complications or die every year from overdoses of cocaine that are not laced with any foreign substance.

This case report details a 40-year-old man who was transported to the ED with altered mental status, agitation, and hyperthermia from suspected cocaine use. He had a history of drug abuse and had multiple hospitalizations in the last year for it. Upon arrival at the ED, he exhibited severe hyperthermia (107.1°F), tachycardia, and tachypnea and was unresponsive to stimuli. Initial lab results indicated significant leukocytosis, metabolic acidosis, rhabdomyolysis, and elevated cardiac troponins. The patient required intubation, aggressive cooling, and intravenous sedation. His clinical course was further complicated by demand ischemia, acute kidney injury, and transaminitis. During his hospitalization, cardiac catheterization ruled out significant coronary artery disease, suggesting that the elevated troponin was due to cocaine-induced vasospasm and myopericarditis. It was subsequently discovered that the patient had a brain lesion previously detected on a computed tomography (CT) scan that was not followed up with magnetic resonance imaging (MRI) by patient request. This lesion required neurosurgical evaluation, and it was concluded that there was no need for acute intervention. Following a long and complex intensive care unit (ICU) stay, the patient was eventually stabilized, extubated, and discharged with outpatient follow-up recommendations.

This case underscores the multifactorial and systemic effects of cocaine toxicity, illustrating the acute dangers and chronic health implications of its use. It highlights the importance of prompt, multidisciplinary management in acute cases and the need for comprehensive long-term care strategies to address the underlying substance use disorder in the outpatient setting. The case adds to the growing body of literature on cocaine-related complications, offering insights into the challenges of managing such patients in a clinical setting.

## Introduction

Cocaine is a powerful stimulant drug that carries significant abuse potential. The overuse and abuse of this substance have been a major public health concern all over the world for decades. It exerts its addictive and euphoric effects primarily by blocking the reuptake of neurotransmitters, specifically norepinephrine, dopamine, and serotonin, leading to an abundance of these chemicals remaining in the synaptic clefts of the central nervous system (CNS) [1]. While cocaine is most significantly associated with the short-lived euphoria it produces, it can lead to long-term and often life-threatening consequences. There is a misnomer that the substance is not damaging in the long term because its effect appears to be short-lived and based on the fact that it only shows up two to three days after use on a urine drug screen. As outlined in this patient presentation, it will be demonstrated that this is surely not the case. Additionally, cocaine is often viewed as only lethal and damaging in the presence of other adulterants like fentanyl, which has recently gained significant attention in the news in discussions surrounding overdose fatality.

Cocaine’s systemic effects are particularly harmful due to its vasoconstrictive and sympathomimetic properties, which can affect multiple organ systems. It has been well documented in the literature that cocaine can cause a variety of neurological, renal, cardiovascular, and metabolic complications. These effects can often be further exacerbated by the drug’s ability to reduce oxygen supply while simultaneously increasing oxygen demand through vasoconstriction and thrombosis [1]. The complications associated with this can range from reversible ischemic injury to end organ failure and, in severe cases, death.

## Case presentation

A male, John Doe, of unknown name and age, presented to the emergency department (ED) via ambulance with a chief complaint of agitation and altered mental status with possible cocaine use. The roommate called because the patient was smashing things around the house. When emergency medical services (EMS) arrived, he was on the bed, agitated and diaphoretic, and on his ride in the ambulance, he became extremely combative. During the ambulance ride, he was administered Versed intramuscularly (IM) and then ketamine IM. On initial arrival to the ED at 12:54, the patient had the vital signs of blood pressure (BP) of 142/92 mmHg, a pulse of 162 beats per minute, a temperature of 107.1°F, and a respiration rate of 41 breaths per minute. On physical exam by EMS, the patient appeared in acute distress, tachycardic, and tachypneic with decreased breath sounds bilaterally. He became unresponsive to EMS verbal stimuli and did not withdraw to painful stimuli. On initial laboratory examination, the patient had a leukocytosis with a left shift (white blood cell (WBC) count of 19.6 with 79.9% absolute neutrophils), with a glucose of 164 and creatinine of 2.04 with hepatitis of aspartate aminotransferase (AST) 206 to alanine transaminase (ALT) of 60. On urinalysis, he had a protein level of 30 mg/dL. On other lab tests, he had a lactic acid of 15.8 mmol/L and a high sensitivity cardiac troponin I of 1,447 ng/L. On arterial blood gas, he had an elevated anion gap acidosis with a pH of 7.18, arterial partial pressure of carbon dioxide (PCO_2_) of 48.1 mmHg, and an arterial bicarbonate (HCO_3_) of 16.8 mmol/L. At 13:00, the patient was intubated for airway protection, put on ventilation with a fraction of inspired oxygen (FiO_2_) of 100%, and weaned down to 40% with positive end-expiratory pressure (PEEP) of five, and an arctic cooling pad was applied after a large amount of ice to the groin, axillary, cervical, and head area. At this time, the patient was bloused with 200 mcg of fentanyl for intravenous (IV) sedation along with a propofol infusion, and FiO_2_ on the ventilator was reduced to 50%.

At this point, the patient was admitted to the ICU, where he had repeat blood gasses drawn at 14:30, which reflected an improvement with a pH of 7.21, PCO_2_ of 45.7, and an arterial HCO_3_ of 18. High sensitivity troponin I was repeated at 17:02 and came back at 2,189, likely due to demand ischemia in the setting of cocaine overdose, rhabdomyolysis, and severe metabolic acidosis. The patient was also started on empiric broad-spectrum antibiotic therapy in the form of IV Vancomycin plus Meropenem to treat bacteremia and sepsis secondary to methicillin-resistant *Staphylococcus aureus* (MRSA), staph, strep, gram-negatives, Pseudomonas, and anaerobes. Over the night, there were periodic drops in the patient’s BP, dropping down to a systolic BP in the 80s. His urine drug screen came back positive for cocaine and cannabinoids, as reflected in Table 1. He was started on a bicarbonate drip containing intravenous fluids (IVF) for his rhabdomyolysis with creatine kinase (CK) of 15,620, with improvement in his renal function to a creatinine of 1.46. At 21:30, code ST-segment elevation myocardial infarction (STEMI) was called, and FiO_2_ was increased to 100% for transfer to another facility for further cardiac evaluation. He arrived by transport at 2236 and was started on a heparin drip in observance of acute coronary syndrome (ACS) protocol. He was transferred to the ICU of this facility for closer cardiac monitoring with the presumptive diagnosis of vasospasm, with a true ST-elevated myocardial infarction being much less likely on the differential diagnosis.

**Table 1:** Urine drug screen (UDS) obtained from the patient on admission, which reflected a positive test for cannabinoids and cocaine THC, tetrahydrocannabinol

Substance	Result
Amphetamines	Negative
Barbiturates	Negative
Benzodiazepines	Negative
THC (cannabinoids)	Positive
Cocaine metabolite	Positive
Opiates	Negative
Phencyclidine	Negative
Methadone	Negative

**Figure 1:**
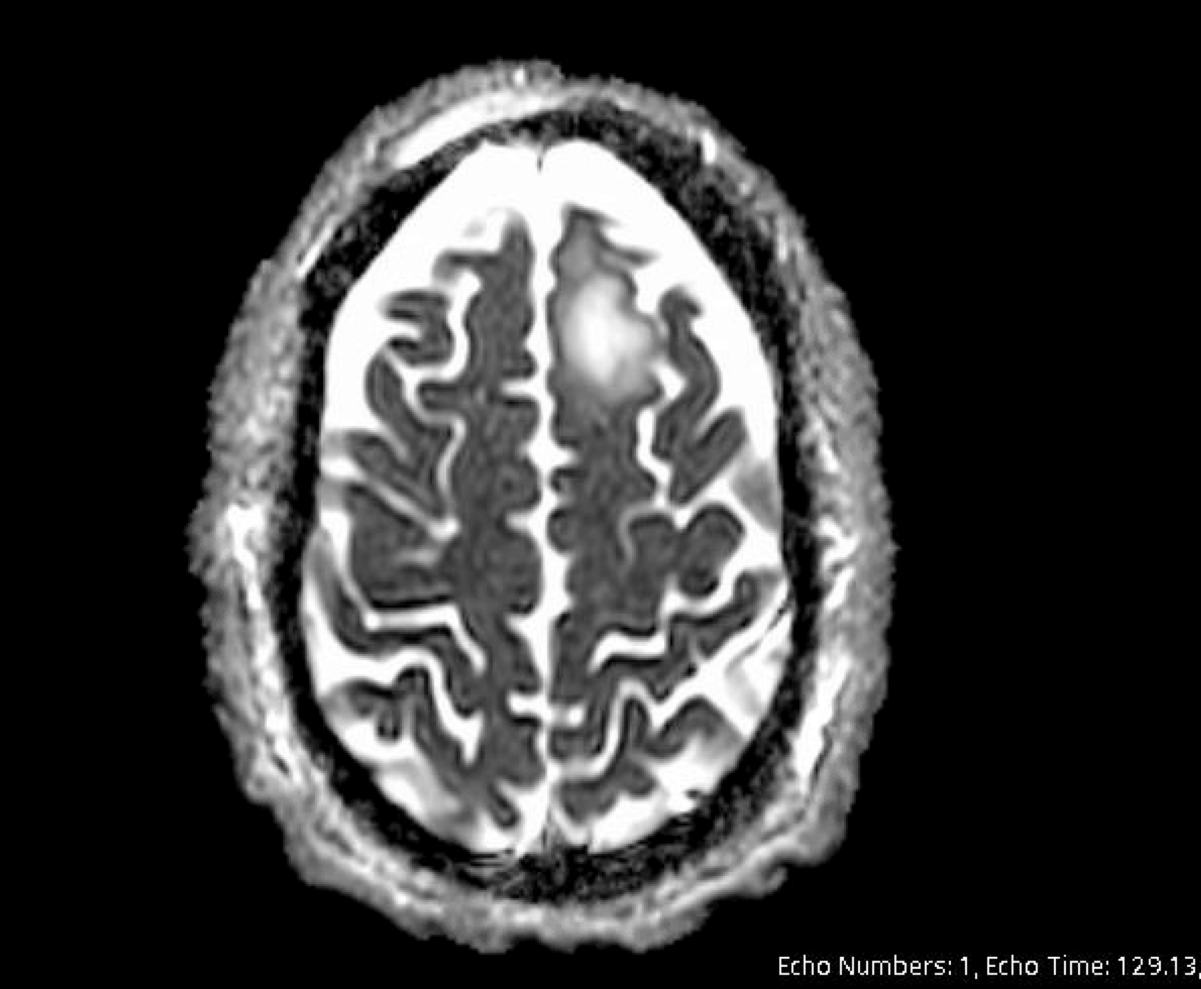
Patient's MRI of the head without contrast demonstrating a left frontal lobe lesion as described above.

Case management was able to identify and meet with the patient’s mother, and it was discovered that he had a history of multiple admissions for overdose. The patient had a head CT done on his second day of hospitalization to rule out cerebral hemorrhage, and it revealed a subtle hypodensity in the left high frontal parasagittal sinus, suggesting possible acute ischemic changes. The lesion was then determined to be present for more than six months now, and it was identified during a previous hospitalization. In the past, it was noted that the patient had left against medical advice (AMA) before he could get an MRI. A subsequent MRI of the head without contrast was performed on day 2 of hospitalization. This scan was significant for a lobular mass-like region of T2 fluid-attenuated inversion recovery (FLAIR) hyperintensity in the anterior left frontal lobe, as seen in the scan in Figure 1. There was no true restricted diffusion with T2 shine, confirmed with the apparent diffusion coefficient (ADC) sequence. This impression yielded a differential of subacute infarct vs. mass, which was indeterminate without contrast. On MRI with contrast, there was no enhancement; concerning an abscess, neurosurgery was consulted. A neurosurgery consult yielded concern for the early stages of an abscess with minimal edema present at this time but with concern about ring formation. With the chronicity of the frontal lobe lesion taken into consideration, there was no intention for acute intervention, and the patient was cleared to follow up with Neurosurgery in the outpatient setting.

**Figure 2:**
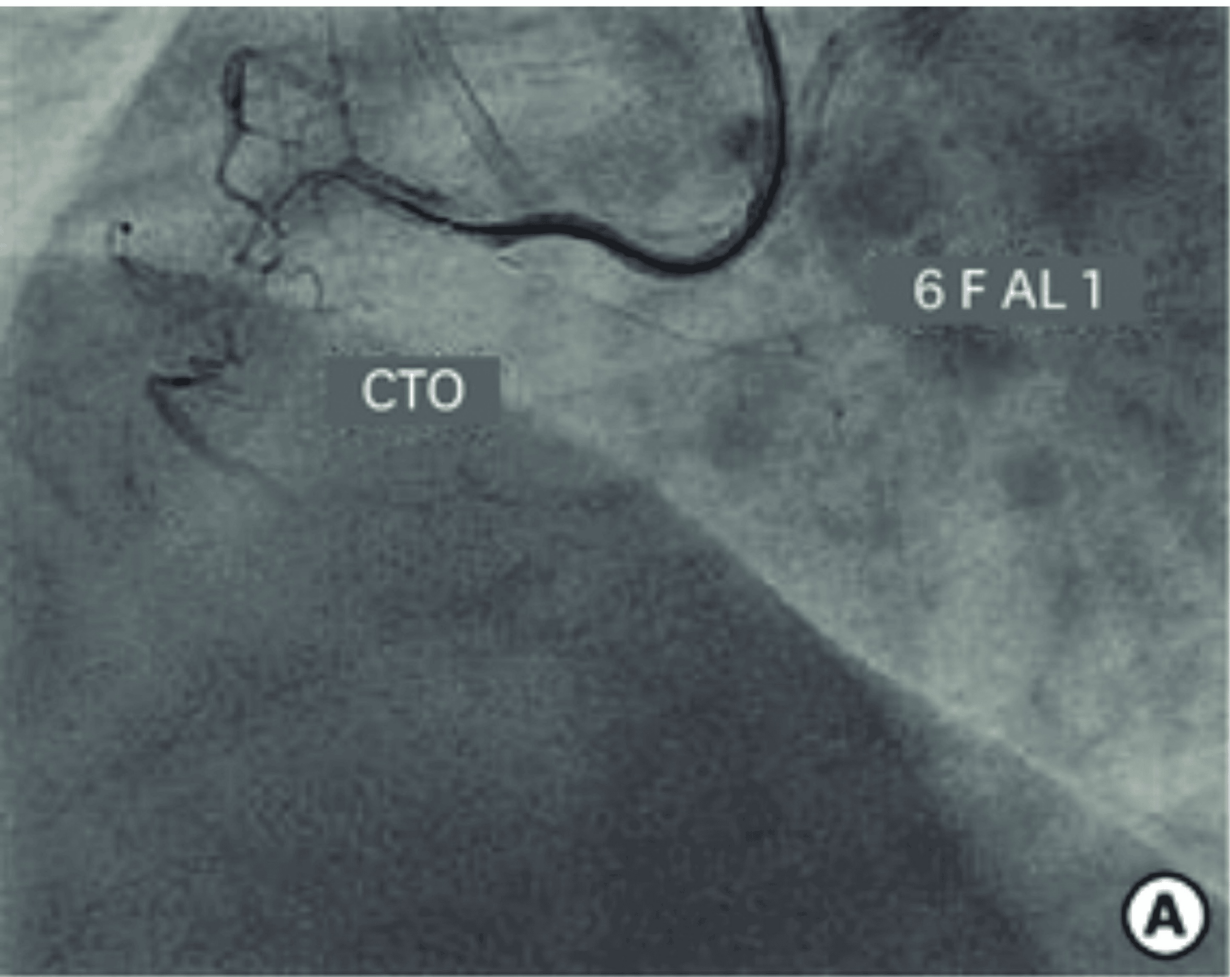
This figure illustrates a catheter engaging the right coronary artery (RCA), which, in the patient described, caused spasm. [2]

Cardiac catheterization was performed and was unremarkable for any coronary artery disease. There was a spasm in the ostial right coronary artery (RCA) on catheter engagement, relived with nitroglycerin without any significant coronary stenosis, as illustrated by the example image from a cardiac catheterization in Figure 2. Normal left ventricular (LV) systolic function and normal LV wall motion were noted. It was concluded that the elevated troponin was not a true non-STEMI and was secondary to demand ischemia in the setting of cocaine use or associated myopericarditis. Heparin drip was discontinued at this time. The patient was cleared for discharge from a cardiac standpoint.

Throughout the last two days of hospitalization, the patient continued to be anxious and impulsive with attempts to pull at the endotracheal (ET) tube. He continued to need bilateral soft wrist restraints to protect his airway and IV placement. On the fourth day of hospitalization, the patient was deemed to be a candidate for extubation. While waiting for resources for extubation, the patient self-extubated without spontaneous breathing trials per patient request. Following extubation, the patient was calm and cooperative and breathing independently.

On social work consultation, the patient reports he was 50 days sober prior to relapse and intends to return to Narcotics Anonymous (NA)/Alcoholics Anonymous (AA) meetings on discharge. On day 2 post-extubation, the patient remained on room air. He was transferred out of the ICU. On day 7 of hospitalization, the patient was deemed a candidate for discharge. Case management reports that the patient does not have health insurance and will be transported home by his family. At the time of discharge, the patient’s rhabdomyolysis had improved with IV fluid. He had transaminitis with an ALT of 467 and an AST of 493, deemed to be likely multifactorial in the setting of rhabdomyolysis, cocaine use, and respiratory failure. He had a negative hepatitis panel. A right upper quadrant ultrasound was performed, which showed a thickened gallbladder wall concerning acalculous cholecystitis. He was cleared from the perspective of general surgery with the recommendation for the patient to follow up as an outpatient regarding possible gallbladder disease with outpatient ultrasound recommended. The patient was recommended to follow up with neurosurgery as an outpatient per his discharge summary.

## Discussion

**Figure 3:**
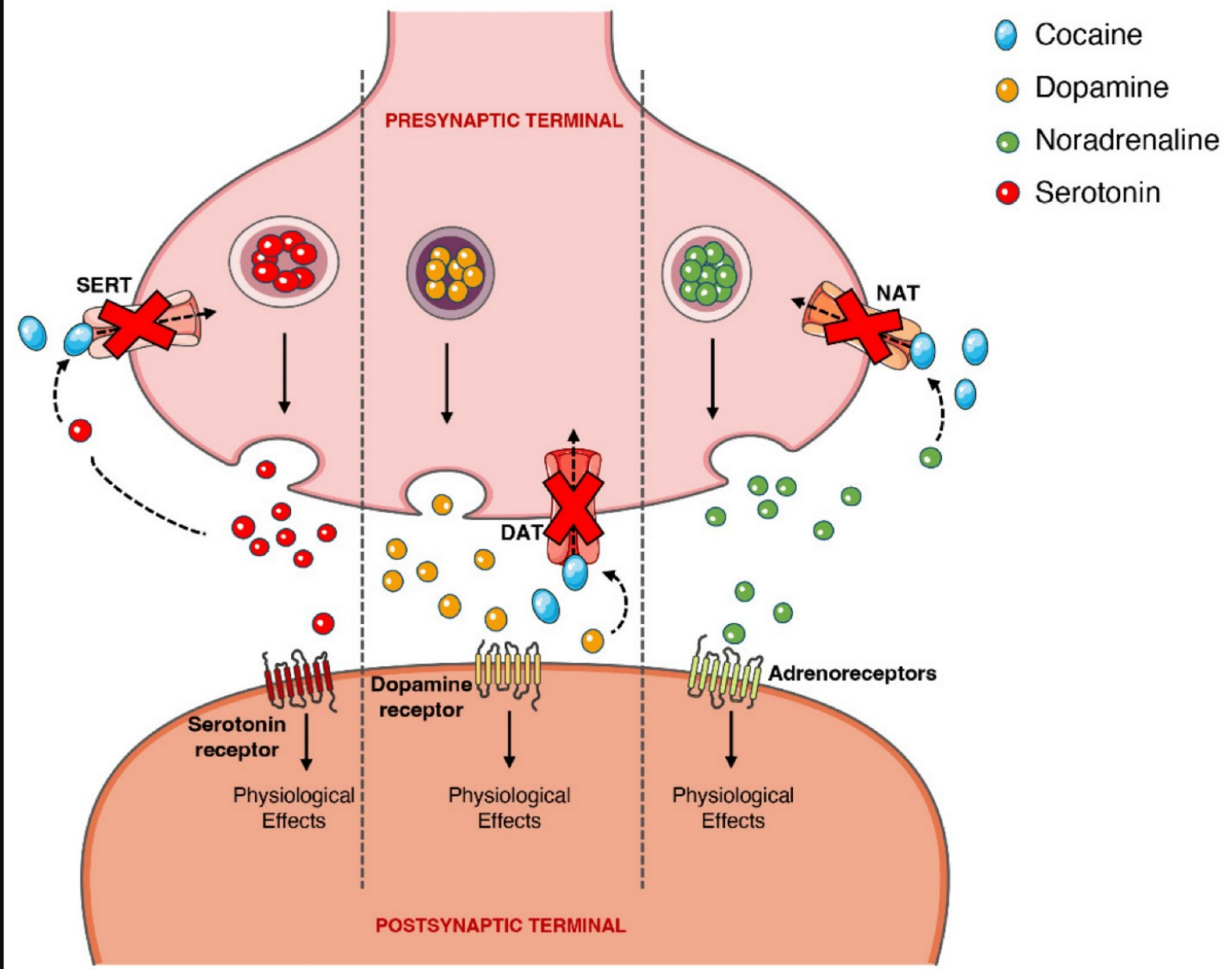
This figure illustrates the effects cocaine has on norepinephrine, dopamine, and serotonin uptake at presynaptic terminals. [1]

Cocaine has the action of blocking conduction in peripheral nerves. This is achieved by reversibly binding to and inactivating sodium channels. Sodium influx through these channels is imperative for the depolarization of nerve cell membranes and the subsequent propagation of impulses along the course of the nerve. Cocaine also possesses vasoconstrictive properties, which are accomplished by blocking norepinephrine in the autonomic nervous system. It binds with more affinity to the serotonin, dopamine, and norepinephrine transport proteins and directly prevents the reuptake of these neurotransmitters into pre-synaptic neurons, as illustrated in Figure 3. The activity of this drug on dopamine levels is most responsible for its addictive properties. This dopamine feedback has been observed specifically in the limbic system of the brain. Specifically, the part of the limbic system called the nucleus accumbens (NAc) appears to be the most important site of the cocaine “high.” When this part of the brain is stimulated by dopamine, cells in the NAc produce feelings of euphoria and a sense of well-being. The utility of this response is to help keep us zoned in on the tasks we are attempting to work on and the basic biological goals of survival and reproduction. When a hungry person eats, for example, dopaminergic cells flood the NAc with dopamine molecules. The response of the receiving cells imparts a feeling of satisfaction and makes us want to repeat the activity and reexperience that pleasure [3].

By artificially causing a buildup of dopamine in the NAc, as described above, cocaine yields enormously powerful feelings of gratification. The amount of dopamine connecting to receptors in the NAc after a dose of cocaine can exceed the amounts associated with natural activities, producing feelings of pleasure that are stronger than that which follow thirst-quenching or sexual activity. In fact, some laboratory animals, if given a choice, will ignore food and keep taking cocaine until they starve [3].

The limbic system also possesses important memory centers located in two regions called the hippocampus and amygdala. These memory centers create memories of what we did that led to the pleasures associated with dopamine release in the NAc. Following that, returning to places where one has taken cocaine triggers emotional responses and desires to repeat the experience. The current literature has shown that repeated cocaine exposure, with its associated dopamine jolts, alters these cells in ways that eventually convert conscious memory and desire into a near-compulsion to respond to cues by seeking and taking the drug, driving the devastating addiction some cocaine users develop [3].

**Figure 4:**
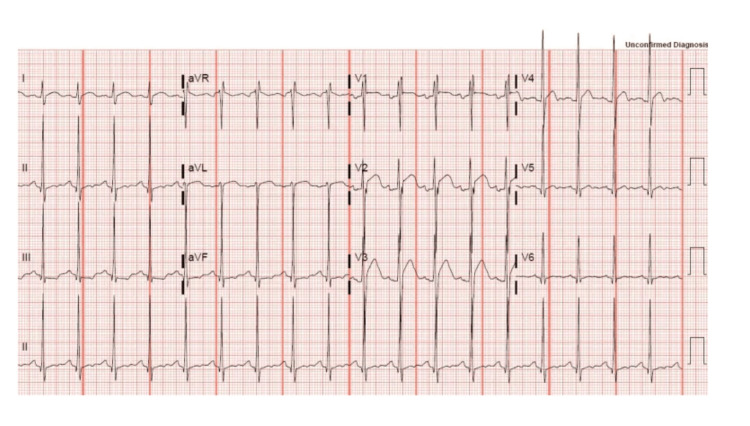
Example of a patient with ST-segment elevations in leads V2 and V3. There are some slight elevations in leads V1 and V4 and minor ST segment depression in leads II, III, and augmented vector foot (aVF). This is a common picture of a patient intoxicated on cocaine due to coronary vasoconstriction. [5]

Cocaine produces a dose-dependent increase in heart rate and BP accompanied by feelings of arousal and mental euphoria; these facts serve as an explanation of why the substance is attractive to many people. Cocaine is known to cause end-organ toxicity in virtually all organ systems, primarily through its hemodynamic effects. To home specifically on one organ system, from a cardiovascular perspective, it is associated with arterial vasoconstriction and enhanced thrombus formation [3]. It increases myocardial oxygen demand and increases vascular shearing forces. This is the driving force behind the concept of “demand ischemia” that is outlined in this case report and reflected in the findings of the example EKG in Figure 4. Cocaine causes coronary vasoconstriction in a dose-dependent fashion and causes cardiac ischemia in 5-6% of patients who come to the emergency room with cocaine-related complaints [4]. Other cardiovascular effects include a negative inotropic effect leading to depressed LV function, precipitating heart failure, as well as supraventricular and ventricular arrhythmias generated through direct actions at myocardial receptors. This negative inotropic effect leading to heart failure can also serve as a contributing factor for the prerenal azotemia observed in this patient’s laboratory studies.

From the perspective of the CNS, cocaine-induced psychomotor agitation can cause hyperthermia when peripheral vasoconstriction prevents the body from dissipating heat generated by persistent agitation [4]. Psychomotor agitation occurs via several mechanisms. Most well documented is when cocaine causes an increase in the CNS excitatory amino acids glutamate and aspartate and release of the excitatory neurotransmitters norepinephrine, serotonin, and dopamine. Additionally, cocaine is associated with both focal neurological deficits and coma. Possible etiologies driving this include cerebral vasoconstriction and intracerebral hemorrhage. There have been many documented cases of patients developing subarachnoid, intraventricular, and intraparenchymal bleeds associated with cocaine use [6].

## Conclusions

This case highlights the profound and widespread impact of cocaine use on the human body, with effects that can lead many organ systems into dysfunction or failure. This patient’s specific presentation of rhabdomyolysis, severe hyperthermia, metabolic acidosis, agitation, altered mental status, and respiratory collapse show how the excessive use of cocaine can quickly take someone’s life. If this patient’s roommate had not heroically and swiftly sought help from emergency services the way he did, we do not believe it is hyperbolic to contend that this patient could have lost his life. Despite aggressive treatment and stabilization efforts, the patient’s course was complicated by cardiovascular, neurological, and hepatic dysfunction, illustrating the complex interplay of direct toxic effects, secondary complications, and pre-existing conditions in patients with cocaine use.

This case underscores the importance of prompt identification of a patient in a drug-intoxicated state and swift multidisciplinary action. While the patient’s hospital management was the major subject matter of this case report, it would be irresponsible not to also discuss the importance of thorough and comprehensive outpatient follow-up. This patient arrived at the hospital with a brain lesion that had been identified eight months prior. At the time, the patient refused further follow-up with an MRI scan and left the hospital AMA. Had this possible abscess been followed up with an MRI at an earlier time, it is theorized that it could have been more effectively characterized and treated. Patient education by case managers and social workers plays a major role in addressing the underlying substance use disorder, aiming to prevent recurrent hospitalizations and improve overall outcomes for patients in similar situations as this gentleman. Through this report, we hope to contribute to the growing body of literature on the multifactorial effects of cocaine, providing insights that may aid in the management and prevention of similar cases in the future.
